# Maternal embryonic leucine zipper kinase enhances gastric cancer progression via the FAK/Paxillin pathway

**DOI:** 10.1186/1476-4598-13-100

**Published:** 2014-05-04

**Authors:** Tao Du, Ying Qu, Jianfang Li, Hao Li, Liping Su, Quan Zhou, Min Yan, Chen Li, Zhenggang Zhu, Bingya Liu

**Affiliations:** 1 Shanghai Key laboratory of Gastric Neoplasms, Shanghai Institute of Digestive Surgery, Department of Surgery, Ruijin Hospital, Shanghai Jiao Tong University School of Medicine, No 197 Ruijin er Road, Shanghai 200025, China

**Keywords:** MELK, Gastric cancer, Tumor migration, Tumor invasion, FAK, Paxillin

## Abstract

**Background:**

Elevated MELK expression is featured in multiple tumors and correlated with tumorigenesis and tumor development. This study is aimed to investigate the mechanisms of MELK-mediated development of gastric cancer.

**Methods:**

MELK expression levels in human gastric cancer were determined by quantitative-PCR and immunohistochemistry. The effect of MELK on cell activity was explored by knockdown and overexpression experiments. Cell growth was measured using the CCK-8 assay. Apoptosis and cell cycle distributions were analyzed by flow cytometry. Migration and invasion were tested using a transwell migration assay. Cytoskeletal changes were analyzed by immunofluorescence. To explore the molecular mechanism and effect of MELK on migration and invasion, Western blotting was used to analyze the FAK/Paxillin pathway and pull down assays for the activity of small Rho GTPases. In vivo tumorigenicity and peritoneal metastasis experiments were performed by tumor cell engraftment into nude mice.

**Results:**

MELK mRNA and protein expression were both elevated in human gastric cancer, and this was associated with chemoresistance to 5-fluorouracil (5-FU). Knockdown of MELK significantly suppressed cell proliferation, migration and invasion of gastric cancer both in vitro and in vivo, decreased the percentages of cells in the G1/G0 phase and increased those in the G2/M and S phases. Moreover, knockdown of MELK decreased the amount of actin stress fibers and inhibited RhoA activity. Finally, knockdown of MELK decreased the phosphorylation of the FAK and paxillin, and prevented gastrin-stimulated FAK/paxillin phosphorylation. By contrast, MELK overexpression had the opposite effect.

**Conclusions:**

MELK promotes cell migration and invasion via the FAK/Paxillin pathway, and plays an important role in the occurrence and development of gastric cancer. MELK may be a potential target for treatment against gastric cancer.

## Background

Gastric cancer (GC) is the fourth most common type of cancer and the second leading cause of cancer deaths worldwide [1,2]. At present, treatment of GC involves surgery, radiotherapy, chemotherapy and molecular targeted therapy [3]. Tumor metastasis and recurrence in patients with GC are considered to be the most significant determinants for treatment failure and mortality [4]. The mechanisms underlying tumor metastasis are very complex, and appear to involve multiple steps [5,6]. There is thus an urgent need to identify the molecular constituents of these mechanisms that could be targeted to improve the treatment of GC.

Maternal embryonic leucine zipper kinase (MELK), a member of the sucrose-non-fermenting (SNF1)/AMPK family of serine-threonine kinases, is a cell cycle dependent protein kinase [7,8]. MELK is conserved across several species including *Xenopus* (pEg3) [9], murine (MPK38) [7] and human (KIAA0175) [10], and plays a key functional role in multiple cellular processes such as the proliferation, cell cycle progression, mitosis, and spliceosome assembly [8,11-15]. Molecularly, MELK interacts with and phosphorylates Ser323 of CDC25B to regulate G2/M progression [8]. The zinc finger protein ZPR9 can also be phosphorylated by MELK to enable its translocation into the nucleus, where it interacts with B-Myb, leading to its increased transcriptional activity [16]. Recent studies also show that MELK is frequently elevated in multiple human tumors such as prostate cancer [17], breast cancer [18], glioblastoma multiforme [19] and medulloblastoma [20], and is correlated with a poor prognosis [21]. Indeed, MELK has recently emerged as an oncogene and a biomarker overexpressed in multiple cancer stem cells [20,22,23], and so is considered a potential therapeutic target [24,25]. Knockdown of MELK inhibited proliferation, colony formation and survival of cancer stem cells [20,26]. In prostate cancers with high Gleason scores, MELK expression was elevated and its inhibition by RNAi detailed putative functions in chromatin modification, embryonic development, and cell migration [17]. In breast cancer, MELK has been found to interact with Bcl-G_L_ through its amino-terminal region and suppress apoptosis [18]. Study also implied that MELK was involved in the resistance of colorectal cancer cells to radiation and 5-FU [27].

The FAK/Paxillin pathway plays an important role in cell migration and invasion [28]. Upon activation of its upstream pathways, FAK binds SH2 domains of Src family kinases, which promotes Src kinase activity through a conformational change and then activates downstream signals to regulate cell motility, invasion, survival and proliferation [29,30]. Activated FAK can phosphorylate various adaptor proteins such as paxillin, which is a multidomain protein located in focal adhesion complexes and connects extracellular matrices to the cytoskeleton [31,32]. The paxillin signaling hub controls the dynamics of focal adhesion assembly and disassembly through protein interactions and phosphorylation events. The FAK/Paxillin pathway also regulates small Rho GTPases, an important family of small GTPases [33]. These proteins, including RhoA, Rac1 and Cdc42, act as molecular switches that cycle between an active GTP-bound and an inactive GDP-bound forms, and play important roles in cytoskeletal reorganization [34]. Paxillin phosphorylation leads to enhanced Rac1 activity and decreased RhoA activity [35,36]. In addition, recent studies have indicated that FAK signaling can promote matrix-degrading invasive behavior through a pathway involving the c-Jun NH2-terminal kinase and MMP-mediated pathways [37].

Here we demonstrate that MELK expression is elevated in tumor-derived primary human gastric tissues compared to normal controls at both mRNA and protein levels. This enhanced expression of MELK is shown to be associated with pleiotropic effects in gastric cancer cells, including increased cell proliferation, migration, and invasion. Finally, we show that MELK can regulate RhoA activity and promote cell migration and invasion via the FAK/Paxillin pathway.

## Results

### MELK is overexpressed in gastric tumor tissues and cell lines

**Figure 1:**
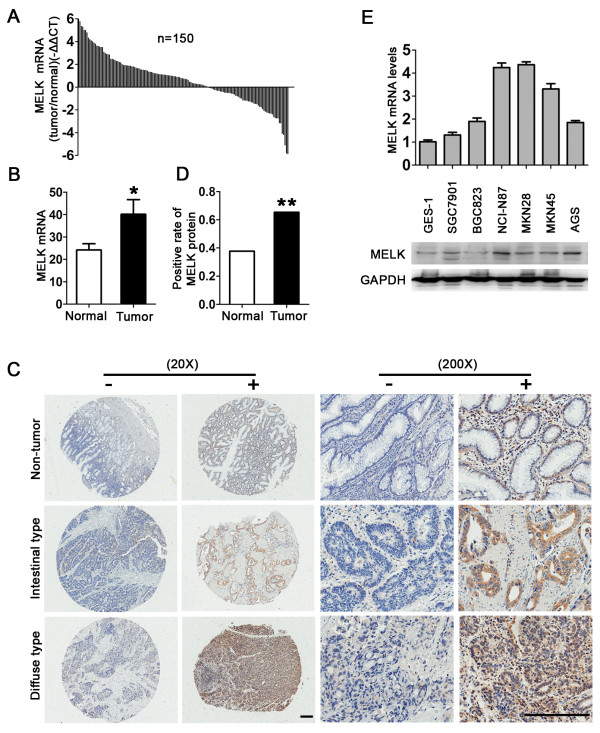
**Elevated MELK expression in gastric cancer tissue array and gastric cancer cells. A** and **B**, Elevated expression of MELK mRNA in 150 pairs of gastric cancer tissues was carried out by qPCR. Data is shown as -ΔΔCt and 2-ΔCt (*P < 0.05). **C**, Expression of MELK was examined by immunohistochemistry staining in non-tumor gastric tissue, diffuse-type gastric cancer and intestinal-type gastric cancer. Original magnification: 20X and 200X. Scale bars = 200 μm. **D**, Analysis of MELK protein expression in 61 non-tumor gastric tissues and 78 gastric cancer tissues (**P < 0.01). **E**, Expression of MELK in human gastric cancer cell lines and immortalized normal gastric cell line. MELK mRNA and protein levels were examined by qPCR and immunoblotting. Data are shown as mean ± SD of three independent experiments.

We evaluated the expression of MELK mRNA in 150 pairs of gastric cancer and non-tumor tissues by qPCR. As shown in Figure 1A and B, we found higher expression levels of MELK mRNA in these gastric cancer tissues compared to non-tumor tissues.

Next we investigated the MELK protein levels in the human gastric cancer and non-tumor tissues by IHC. Ninety-eight pairs of tumor and non-tumor gastric tissues were stained, and the MELK protein was found to be localized in the cytoplasm. The positive rates of MELK protein detection were 37.7% (23/61) in non-tumor tissues and 65.4% (51/78) in tumor tissues, which is significant (Figure 1C and D). Furthermore, statistical analysis showed that the presence of the MELK protein was significantly correlated with clinicopathological parameters. As shown in Table 1, the presence of MELK was elevated in well differentiated and intestinal type gastric cancer. We also found that MELK mRNA and protein were elevated in gastric cancer cell lines compared with the immortalized normal gastric epithelial cell line GES-1 (Figure 1E). Together, these data clearly indicate that MELK is overexpressed in gastric cancer tissues and cell lines.

**Table 1:** Relationship between MELK expression level and clinicopathologic variables in 78 gastric cancer tissues

**Clinicopathologic parameters**	**MELK protein**		**P**
	**−(n = 27)**	**+(n = 51)**	
Age (years)			
≤60	12	23	0.522
>60	15	21	
Gender			
Male	21	38	0.749
Female	6	13	
Tumor size (cm)			
≤5	17	33	0.879
>5	10	18	
Lauren classification			
Intestinal	2	25	<0.001
Diffuse	25	26	
Differentiation			
Poorly, undifferentiated	25	31	0.003
Well, moderatelly	2	20	
Local invasion			
T1,T2	6	14	0.615
T3,T4	21	37	
Lymph node metastasis			
No	3	14	0.096
Yes	24	37	
TNM stage			
I,II	5	12	0.61
III,IV	22	39	

### MELK is associated with resistance of gastric cancer cells to 5-FU

**Figure 2:**
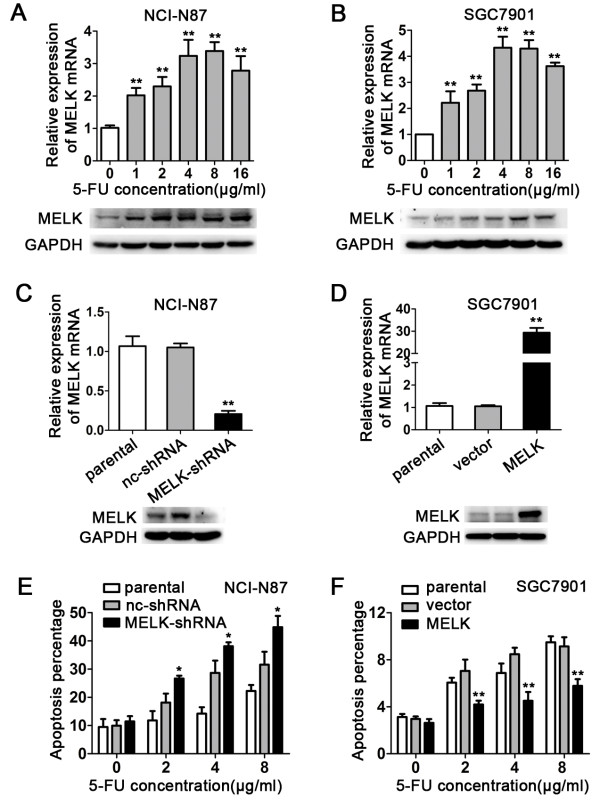
**MELK is associated with resistance of gastric cancer cells to 5-FU. A** and **B**, Effect of 5-FU on MELK expression level (**P < 0.01). NCI-N87 and SGC7901 cells were treated with 0, 1, 2, 4, 8, 16 μg/ml of 5-FU, and MELK expression was examined by qPCR and Western blotting 48 h later. **C** and **D**, qPCR and immunoblotting analysis of the efficacy of MELK knockdown and overexpression (**P < 0.01). pGPU6/GFP/Neo was used for shRNA plasmid construction. Plasmids were transfected into gastric cancer cells using Lipofectamine 2000. MELK cDNA ORF was cloned into pL/ERES/GFP plasmid for lentivirus production. **E** and **F**, Effect of MELK knockdown and overexpression on apoptosis induced by 5-FU. Cells were treated with 0, 2, 4, 8 μg/ml of 5-FU, apoptosis was examined by flow cytometry (*P < 0.05, **P < 0.01).

In order to investigate the relationship between MELK and chemoresistance to 5FU in gastric cancer cells, SGC7901 and NCI-N87 cells were exposed to different concentrations of 5-FU (0, 1, 2, 4, 8, 16 μg/ml), and cells were then collected after 48 h for qPCR analysis and Western blotting to determine the MELK expression level. We found that the expression level of MELK in these two cell lines significantly increased after 5-FU treatment (Figure 2A and B). To further explore the effect of MELK on chemoresistance to 5FU, we used shRNA to generate MELK-knockdown NCI-N87 cells (NCI-N87/MELK-shRNA) and used the pL/ERS/GFP lentivirus vector to generate MELK-overexpressing SGC7901 cells (SGC7901/MELK). The efficacy of MELK knockdown and overexpression is shown in Figure 2C and D. More than 80% of MELK mRNA and protein was suppressed in the NCI-N87 cells compared with the negative control (NCI-N87/nc-shRNA) cells, while these were dramatically increased in SGC7901 cells compared with the empty vector transfected (SGC7901/vector) cells.

To investigate whether MELK is associated with cell apoptosis induced by 5-FU, NCI-N87/MELK-shRNA, SGC7901/MELK, and control cells were treated with different concentrations of 5-FU (0, 2, 4, 8 μg/ml) followed by analysis for apoptosis. Apoptosis was determined by flow cytometry (FCM) 48 h after adding 5-FU. Compared with the control group, apoptosis of the MELK-knockdown group gradually increased in a manner that correlated with the 5-FU concentration (Figure 2E and Additional file 1: Figure S1-A). In contrast, apoptosis decreased proportionally to the 5-FU concentration in the SGC7901/MELK group (Figure 2F and Additional file 1: Figure S1-B). These data indicated that MELK could be associated with resistance of gastric cancer cells against 5-FU.

### MELK is involved in cell proliferation, cell cycle progression, migration, and invasion

To further investigate the cellular effects of MELK, we evaluated cell proliferation, cell cycle progression, migration, and invasion of NCI-N87/MELK-shRNA and SGC7901/MELK cells. We analyzed cell proliferation with the CCK-8 (Cell Counting Kit-8) assay and found that the knockdown of MELK reduced cell proliferation compared to the control cells, and that MELK overexpression slightly promoted cell proliferation (Additional file 2: Figure S2-A and S2-B). Next we analyzed the cell cycle distribution by FCM. The percentage of cells located in the G2/M and S phase was higher in NCI-N87/MELK-shRNA than control and parental cells, and the percentage in the G1/G0 phase was lower (Additional file 2: Figure S2-C and S2-D). Interestingly, MELK overexpression also increased cell populations in the G2/M and S phases and decreased those in the G1/G0 phase (P < 0.05) (Additional file 2: Figure S2-E and S2-F).

**Figure 3:**
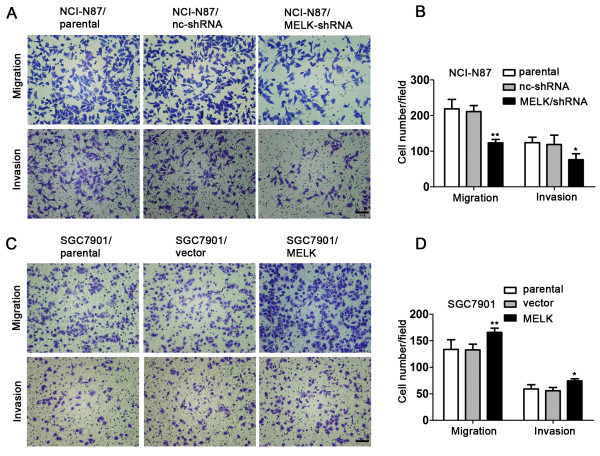
**Effects of MELK knockdown and overexpression on cell migration and invasion *in vitro*. A** and **B**, NCI-N87 cell migration and invasion were analyzed by a transwell chamber assay (100X) (*P < 0.05, **P < 0.01). Scale bars = 100 μm. **C** and **D**, SGC7901 cell migration and invasion. These data are shown as mean ± SD of three independent experiments.

We next examined cell migration and invasive ability with a transwell migration, transwell invasion, and wound healing assays. The amount of migrated and invaded cells in NCI-N87/shRNA group was significantly decreased compared with the control cells (Figure 3A and B). In contrast, the SGC-7901/MELK group was moderately increased compared with the control cells (Figure 3C and D). In the wound healing assay, NCI-N87/nc-shRNA cells nearly closed the wound 72 h after scratching, whereas NCI-N87/shRNA cells were unable to heal the wound (Additional file 3: Figure S3-A). The wound areas of the experimental group and controls were significantly different (Additional file 3: Figure S3-C). However, MELK overexpression promoted SGC7901 wound healing (P < 0.05) (Additional file 3: Figure S3-B and S3-D). Thus, together, these results implicate MELK in the regulation of characteristic cellular behaviors found in gastric cancers.

### MELK affects cell morphology and the cytoskeleton

**Figure 4:**
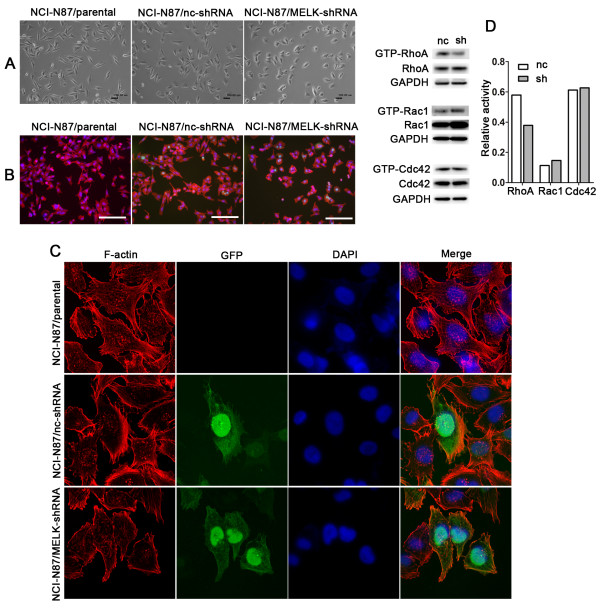
**Effects of MELK knockdown on the cytoskeleton and small Rho-GTPase activity. A**, Images of NCI-N87/MELK-shRNA and control cells (100X). Scale bars = 200 μm. **B**, Immunostaining of phalloidin (F-actin) in NCI-N87/MELK-shRNA and control cells (100X). Red: F-actin; Blue: DAPI. Scale bars = 50 μm. **C**, Immunostaining of phalloidin (F-actin) and DAPI (nucleus) using confocal microscopy (400X). **D**, Small Rho-GTPase activity in NCI-N87/MELK-shRNA and control cells was measured by a Rhotekin-RBD or PAK-PBD pulldown assay. Data shows examples taken from one of three independent experiments.

We first observed dramatic cell morphological alterations in cells both MELK knockdown and overexpression. NCI-N87 cells transfected with MELK-shRNA rendered smaller size and fewer filopodia (Figure 4A). In contrast, SGC7901 cells exhibited larger size when MELK was overexpressed (Additional file 4: Figure S4-A). We then asked whether MELK expression had an effect on the cytoskeleton. To do so, cells were stained for F-actin by Immunofluorescence (IFC). As we expected, we also detected similar cell morphological alterations (Figure 4B and Additional file 4: Figure S4-B). In addition, analysis of the cytoskeleton by confocal microscopy indicated that MELK knockdown significantly reduced the number of actin stress fibers and filopodia, while MELK overexpression increased the occurrence of actin stress fibers (Figure 4C and Additional file 4: Figure S4-C).

### MELK inhibits RhoA activity

As small Rho-GTPases play important roles in cytoskeleton regulation, we next analyzed the effect of MELK expression on Rho-GTPases (RhoA/Rac1/Cdc42). Rho-GTPase activities were measured using the Rho GTPases activation Assay Combo Biochem Kit according to the manufacturer’s instructions. Knockdown of MELK significantly inhibited RhoA activity but had no effect on Rac1 and Cdc42 activity (Figure 4D). In contrast, MELK overexpression slightly promoted RhoA activity and had no effect on Rac1 and Cdc42 (Additional file 4: Figure S4-D).

### MELK promotes FAK and paxillin phosphorylation

**Figure 5:**
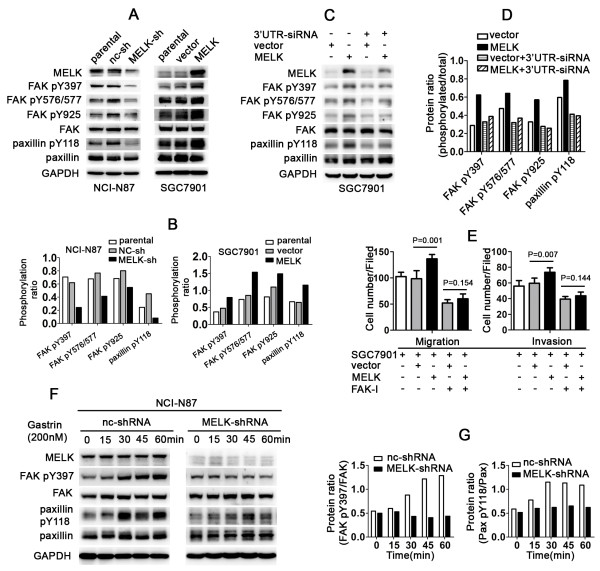
**MELK could regulate cell migration and invasion via the FAK/Paxillin pathway. A** and **B**, Effects of MELK on FAK and paxillin phosphorylation levels. Cellular lysates were analyzed by immunoblotting. **C** and **D**, 3′UTR-siRNA of MELK partially reversed the up-regulation of pY397, pY576/577, and pY925 of FAK, and pY118 of paxillin caused by MELK overexpression. SGC7901/vector and SGC7901/MELK cells were transfected with 3′UTR-siRNA of MELK using Lipofectamine 2000; cells were collected and immunoblotting was performed 48 h later. **E**, Effect of FAK inhibitor (FAK-I) on SGC7901 cell migration and invasion. SGC7901, SGC7901/vector and SGC7901/MELK cells were treated with FAK inhibitor (10 μM); cell migration and invasion were measured after 2 h. **F**, Knockdown of MELK prevents gastrin-stimulated FAK and paxillin phosphorylation. NCI-N87/NC-shRNA and NCI-N87/MELK-shRNA cells were treated with gastrin (200nM) and cells were collected and analyzed by immunoblotting after 0, 15, 30, 45, 60 mins. **G**, Ratio of phosphorylated to total protein, respectively. (Pax: paxillin).

FAK/Paxillin pathway is involved in cell migration and that paxillin can regulate the activities of Rho-GTPases. We thus explored whether MELK levels affected the phosphorylation levels of FAK/paxillin. As shown in Figure 5A and B, MELK knockdown significantly inhibited Tyr397, Tyr576/577, and Tyr925 phosphorylation of FAK and Tyr118 phosphorylation of paxillin. In contrast, MELK overexpression enhanced phosphorylation of these proteins. This indicated that MELK might be involved in the regulation of the FAK/Paxillin pathway as an upstream molecule. Next we examined whether MELK knockdown could reverse the up-regulation of phosphorylation caused by MELK overexpression. We designed two siRNAs that target the 3′untranslated region (3′UTR) and coding sequence region (CDS) of MELK. SGC7901/MELK and SGC7901/vector cells were transfected with siRNAs and then analyzed by Western blotting after 48 h. We found that both MELK siRNAs indeed partially reversed the up-regulation of Tyr397, Tyr576/577, and Tyr925 phosphorylation of FAK and Tyr 118 phosphorylation of paxillin (Figure 5C-D and Additional file 5: Figure S5A-5B).

### MELK regulates cell migration and invasion via the FAK/Paxillin pathway

As the aforementioned results showed that MELK promotes FAK phosphorylation, and it is well known that FAK is an important regulator of cell migration and invasion, we speculated that MELK could regulate cell migration and invasion via the FAK/Paxillin pathway. We treated SGC7901 cells with a FAK inhibitor (10 μM) and analyzed cell migration and invasion. We found that the differences between SGC7901/MELK and SGC7901/vector cells in migration and invasion were significantly decreased after treatment with the FAK inhibitor (Figure 5E and Additional file 6: Figure S6; P = 0.001 vs. 0.154 and P = 0.007 vs. 0.144). This result indicates that the inhibition of FAK can counter-act the up-regulatory effect on migration and invasion caused by MELK overexpression. Thus, these data combined indicate that MELK regulates cell migration and invasion via the FAK/Paxillin pathway.

### MELK knockdown prevents gastrin-stimulated FAK and paxillin phosphorylation

Gastrin plays an important role in the development of multiple tumors and induces FAK and paxillin phosphorylation, suggesting that this might be a factor of tumor pathogenesis [38]. We thus examined whether knockdown of MELK could prevent gastrin-stimulated FAK and paxillin phosphorylation. NCI-N87/MELK-shRNA and NCI-N87/nc-shRNA cells were treated with gastrin (200 μM) and cells were collected and analyzed by Western blotting after 0, 15, 30, 45, and 60 mins. Indeed, Tyr397 phosphorylation of FAK and Tyr118 phosphorylation of paxillin were gradually increased in NCI-N87/nc-shRNA cells in a time-dependent manner. However, there was no significant alteration in NCI-N87/shRNA cells (Figure 5F and G). These data indicate that MELK prevents gastrin-stimulated FAK and paxillin phosphorylation.

### MELK specific inhibitor OTSSP167 suppresses cell migration and invasion

**Figure 6:**
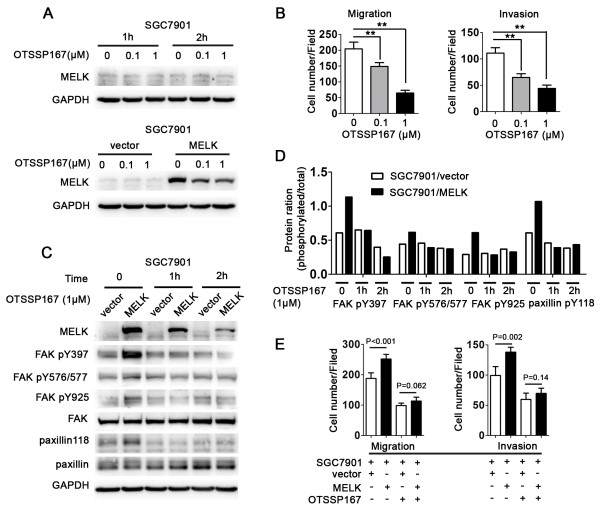
**MELK specific inhibitor OTSSP167 suppresses cell migration and invasion. A**, Immunoblotting representing MELK expression in SGC7901 cell following OTSSP167 treatment for indicated period. In the upper panel, SGC7901 cell were treated with different concentration (0, 0.1, 1 μM) of OTSSP167 for 1 h and 2 h. In the lower panel, SGC7901/vector and SGC7901/MELK cell were treated with 1 μM OTSSP167 for 2 h. **B**, OTSSP167 suppresses SGC7901 cell migration and invasion measured by a transwell chamber assay (**P < 0.01). **C** and **D**, OTSSP167 partially reverses the up-regulation of pY397, pY576/577, and pY925 of FAK, and pY118 of paxillin caused by MELK overexpression. SGC7901/vector and SGC7901/MELK cells were treated with 1 μM OTSSP167 for indicated period and then were collected and measured by immunoblotting. **E**, Effect of OTSSP167 on SGC7901 cell migration and invasion. SGC7901/vector and SGC7901/MELK cells were treated with OTSSP167 (1 μM); cell migration and invasion were measured after 2 h.

OTSSP167, a specific inhibitor of MELK, is recently reported to suppress MELK expression and/or activity [25]. We therefore investigated whether or not pretreatment with OTSSP167 on SGC7901 cells resulted in a reduction of cell migration and invasion. We incubated SGC7901, SGC7901/MELK and SGC7901/vector with different concentrations of OTSSP167 (0, 0.1, 1 μM) for 1 h or 2 h. MELK expression was measured by immunoblotting. Interestingly, OTSSP167 slightly reduced expression of MELK protein in SGC7901 and SGC7901/vector, but significantly reduced MELK expression in SGC7901/MELK (Figure 6A). Next we examined cell migration and invasive ability. As shown in Figure 6B, OTSSP167 significantly suppressed cell migration and invasion. Furthermore, similar to MELK-siRNA, OTSSP167 partially reversed the up-regulation of Tyr397, Tyr576/577, and Tyr925 phosphorylation of FAK and Tyr 118 phosphorylation of paxillin (Figure 6C and D), as well as the up-regulatory effect on migration and invasion (Figure 6E; P < 0.001 vs. 0.062 and P = 0.002 vs. 0.14) caused by MELK overexpression.

### MELK promotes tumor growth, peritoneal spreading and metastasis in vivo

**Figure 7:**
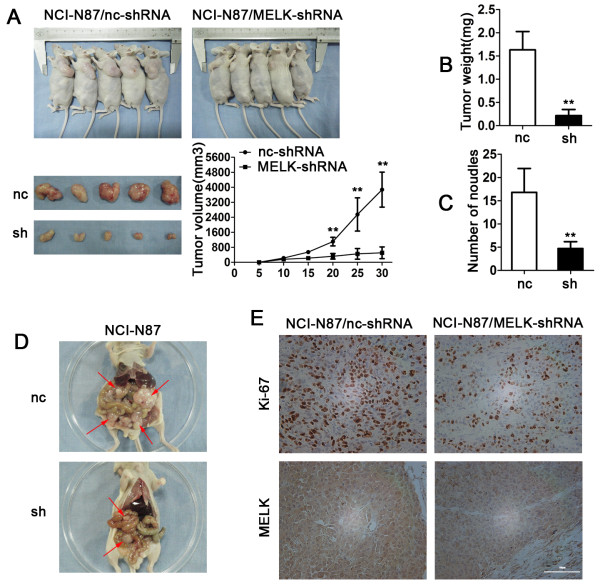
**Effects of MELK on tumor growth, peritoneal spreading and metastasis in vivo. A**, Photographs of tumors derived from NCI-N87/nc-shRNA and NCI-N87/MELK-shRNA cells and growth curves of tumors in nude mice (*P < 0.05; n = 5 per group). Tumor diameters were measured every 5 days. **B**, Average weights of tumors in nude mice (**P < 0.01). **C** and **D**, Effects of MELK knockdown on peritoneal spreading and metastasis (**P < 0.01; n = 10 per group). Metastatic nodules were obvious in the control group as indicated by the red arrows. **E**, Representative photographs of immunohistochemical analysis of Ki-67 antigen and MELK protein in tumors of nude mice (original magnification, 200X).

Finally, we tested whether MELK can regulate tumor growth and peritoneal spreading and metastasis. NCI-N87/MELK-shRNA, NCI-N87/nc-shRNA, SGC7901/MELK and SGC7901/vector cells were subcutaneously or intraperitoneally injected into the nude mice. We found that MELK knockdown inhibited tumor growth (Figure 7A). Tumor weights were less in the NCI-N87/MELK-shRNA group compared with the NCI-N87/nc-shRNA group (0.21 ± 0.13 g vs. 1.63 ± 0.39 g, P < 0.01, Figure 7B). Furthermore, the peritoneal nodules were less in the NCI-N87/MELK-shRNA group compared with the NCI-N87/nc-shRNA group (4.7 ± 1.49 vs. 16.8 ± 5.13, P < 0.01, Figure 7C and D). In contrast, MELK overexpression promoted tumor growth (1.6 ± 0.50 g vs. 0.54 ± 0.14 g, P < 0.01) and peritoneal spreading and metastasis (22.3 ± 7.06 vs. 14.9 ± 3.38, P < 0.01, Additional file 7: Figure S7A-7D). However, the effect of MELK knockdown on tumor growth and metastasis was more profound than MELK overexpression. We also analyzed the expression of Ki-67 antigen by IHC, a cellular mark for proliferation. The number of Ki-67-antigen-positive cells and staining intensity were significantly lower in the tumors derived from the NCI-N87/MELK-shRNA cells compared to the NCI-N87/nc-shRNA cells, and were slightly higher in the SGC7901/MELK group compared with the SGC7901/vector control (Figure 7E and Additional file 7: Figure S7-E). These results suggest that MELK promotes tumor growth, peritoneal spreading and metastasis in vivo.

## Discussion

Recent studies have shown that MELK plays an important role in tumorigenesis and tumor development [8,12,17,24]. However, the exact mechanism has not been established. Here, we showed that MELK expression was up-regulated in gastric tumors. MELK knockdown and overexpression models demonstrated its role in regulating cell proliferation, cell cycle progression, and chemoresistance to 5FU. Furthermore, we observed that MELK regulated the activity of RhoA and promote cell migration and invasion via the FAK/Paxillin pathway.

Many studies have shown that MELK is highly expressed in tumors and this expression is correlated with tumor grade and prognosis. For example, Gray et al. examined 3600 normal and 1701 cancer tissues by oligonucleotide microarray analysis, including breast, cervix, colorectal, esophagus, kidney, liver, and ovary cancers [24]. Although MELK has been the focus of many cancer-related studies, most of these lacked data related to the protein level, and few have investigated gastric cancer. We examined here MELK expression in clinical tissue samples and cell lines, and found that MELK mRNA and protein expression were both elevated in tumor tissues. We also analyzed the correlation between MELK expression and clinicopathological parameters and found that MELK protein expression was higher in well differentiated and intestinal type gastric cancers.

Previous studies also suggested that MELK expression was elevated in cancer stem cells (CSCs) and could promote CSCs growth, differentiation and self-renewal [11,23,39,40]. In agreement with this earlier work, we found that MELK had wide-spread effects involving chemoresistance, cell proliferation, migration, invasion and cytoskeleton regulation in gastric cancer cells. We speculate that this is owing to the effect of MELK on gastric CSCs. In particular, knockdown of MELK dramatically suppressed tumor growth in vivo, although the effect was not so significant in vitro. Besides blocking the cell cycle, the main reason could be that MELK suppressed the proliferation of gastric CSCs. We also found that MELK expression was elevated after treatment with different concentrations of 5-FU, and MELK could regulate apoptosis induced by 5-FU. As chemoresistance is closely correlated with CSCs and MELK is generally regarded as a marker of CSCs, we propose that MELK expression might be elevated in gastric CSCs and is closely related to chemoresistance in gastric cancer. We also hypothesize that MELK might be a potential target for chemotherapy, but further study is needed to support this. Furthermore, it appeared as though the effect of MELK overexpression was not as dramatic as its knockdown. This might be due to the already relative high expression of MELK in gastric cancer. Interestingly, MELK knockdown and overexpression both resulted in increased cell populations in the G2/M phase. Several studies also found these contradictory results [8,24,41]. In these, it was suggested to be owing to a delay of G2/M. In addition, MELK might be necessary for cells to through a G2/M checkpoint but its expression and activity are both suppressed once the cell has overcome this checkpoint.

The migration and invasion of cancer cells involves a host of processes and the interaction of multiple genes [42]. Previous studies indicated that MELK plays an important role in tumors, but the mechanism was unclear. In this study we found that MELK knockdown or overexpression decreased or increased Tyr397, Tyr576/577, and Tyr925 phosphorylation of FAK and Tyr118 phosphorylation of paxillin, respectively. The FAK inhibitor and MELK inhibitor could both partly reverse the up-regulatory effect on migration and invasion caused by MELK overexpression. This indicated that MELK could be an upstream regulator of FAK. Furthermore, we found that MELK prevented gastrin-stimulated FAK and paxillin phosphorylation, which may be an important factor for tumorigenesis. These data strongly support the role of MELK in regulating cell migration and invasion via FAK/Paxillin pathway.

As MELK could regulate the phosphorylation of paxillin, we also analyzed its effect on the cytoskeleton by IFC. We found that MELK knockdown decreased the amount of actin stress fibers and filopodia, while MELK overexpression resulted in an increase in these structures. We speculated that it might be due to the elevation of Cdc42 and RhoA activity. The phosphorylation of FAK Tyr397 can promote Rac1 activity via the Crk/Dock180/ELMO complex [33,43]. Furthermore, phosphorylated Tyr31/Tyr118 of paxillin can bind to p120RasGAP, which releases the inhibitory interaction of p120RasGAP with p190RhoGAP and then suppresses RhoA activity [36]. However, we found that knockdown of MELK inhibited RhoA activity, whereas its overexpression promoted RhoA activity. In addition, there was no effect on the activities of Rac1 and Cdc42, which seemed rather contradictory. Similarly, Zhai [44] also found that the overexpression of FAK increased RhoA activity and p190RhoGEF phosphorylation in neuronal cells. Our data thus provide additional evidence that FAK could have both positive and negative effects on RhoA. Thus, the effect of MELK through RhoA or FAK/paxillin may affect cell migration and invasion.

In summary, our data indicate that MELK expression is elevated in gastric cancer. MELK plays an important role in the regulation of cell proliferation, cell cycle progression, chemoresistance, migration, invasion, and cytoskeleton assembly. Furthermore, MELK was found to promote cell migration and invasion via the FAK/Paxillin pathway, which could thus be a potential focus of future therapy against gastric cancer.

## Methods

### Ethical statement

Written informed consent was obtained from all participants. The study was approved by the Human Research Ethics Committee of Ruijin Hospital, School of Medicine, Shanghai Jiao Tong University (Permit number: HREC 08–028). All animal experiments were approved by the Laboratory Animal Ethics Committee of Ruijin Hospital (Permit Number: 2013062) and performed in accordance with the Guide for the Care and Use Laboratory Animals of Ruijin Hospital, School of Medicine, Shanghai Jiao Tong University.

### Cell lines and chemicals

Gastric cancer cell lines SGC7901, BGC823, MKN45, MKN28, NCI-N87, AGS and an immortalized normal gastric epithelial cell line GES-1 were preserved in the institute. Cells were cultured in RPMI-1640 medium containing 10% fetal calf serum with 100U/ml penicillin and 100ug/ml streptomycin (GIBCO BRL) and maintained at 37°C in a humidified atmosphere of 5% CO_2_. FAK inhibitor and gastrin were from Millipore. MELK inhibitor OTSSP167 was from MedChem Express.

### Tissues

Gastric cancer tissues were obtained from 150 patients who underwent radical gastrectomy between 2006 and 2008 at the Department of Surgery, Ruijin Hospital, Shanghai, China. All samples were confirmed by pathological diagnosis. All tissue samples were formalin-fixed and paraffin-embedded. Eighty pairs of tissue samples from patients were processed into tissue arrays and confirmed by a pathologist.

### Plasmids construction and transfection

For MELK knockdown, the target sequence was 5′-GGATCTCAACCAAGCACATAT-3′, the negative control sequence was 5′-GTTCTCCGAACGTGTCACGT-3′. pGPU6/GFP/Neo (GenePharma) was used for shRNA plasmid construction. Plasmids were transfected into gastric cancer cells using Lipofectamine 2000 (Invitrogen). For 3′UTR of MELK knockdown, the target sequence was 5′-GCCTACATAAAGACTGTTA-3′, the negative control sequence was 5′-GTTCTCCGAACGTGTCACGT-3′. MELK cDNA ORF (Origene Technologies) was cloned into the pL/ERES/GFP plasmid (Novobio) for lentivirus production.

### qPCR (quantitative-PCR)

Total RNA was isolated using Trizol reagent (Invitrogen) and cDNA was obtained using a reverse transcription kit (Promega) according to the manufacturer’s instructions. qPCR was performed using the Applied Biosystems 7900HT sequence detection system (Applied Biosystems) and Universal PCR Master Mix (Applied Biosystems). Relative expression was calculated with GAPDH using the 2^-ΔCt^ and -ΔΔCt method. The primers for MELK were 5′-CATTAGCCCTGAGAGGCGGTGC-3′ (fwd) and 5′-GCCCGTCTCTGGCAGAACCCTT-3′ (rev). The primers for GAPDH were 5′-TTGGCATCGTTGAGGGTCT-3′ (fwd), and 5′-CAGTGGGAACACGGAAAGC-3′ (rev).

### Immunohistochemistry staining

Immunohistochemistry (IHC) staining was performed as previously reported [45]. Polyclonal anti-MELK was used at a dilution of 1:150 (Sigma). The slides were evaluated by a single board-certified pathologist (RRT) without clinicopathologic information. The percentage of positive cells was divided into five grades (percentage scores): <5% (0), 5-25% (1), 25-50% (2), 50-75% (3), 75-100% (4). The intensity of staining was divided into four grades (intensity scores): no staining (0), weak staining (1), moderate staining (2) and strong staining (3). MELK staining positivity was determined by the following formula: overall score = percentage score × intensity score. The overall score ≤ 3 was defined as negative, and >3 as positive.

### Immunoblotting

Cells were lysed using RIPA cell lysis buffer (Kangwei) supplemented with protease inhibitor cocktail (Cell Signaling Biotechnology). The amount of total protein was quantified using a protein assay kit (Bio-Rad). Protein samples were loaded onto 12.5% SDS-PAGE gels and then transferred onto PVDF membranes. The membranes were blocked in TBS-T buffer containing 5% non-fat dry milk and hybridized with a primary antibody. Paxillin (Tyr118) antibody was from Abcam, GAPDH antibody was from Kangchen Bio-tech, and all other primary and secondary antibodies were from Cell Signaling Biotechnology. Finally, membranes were incubated with HRP-conjugated secondary antibody. Protein bands were visualized using ECL reagent (Thermo) on a Tanon detection system.

### Cell proliferation assay

Cell proliferation was assayed using Cell Counting Kit-8. Cells were cultured in a 96-well plate at a concentration of 1 × 10^4^ cells/ml; OD450 was measured 2 h after adding CCK-8 at 0, 1, 2, 3 and 4 days.

### Flow cytometry

For cell cycle analysis, cells were harvested and fixed in 70% ice-cold ethanol at 4°C overnight and then incubated with 100 μg/ml RNase at 37°C for 20 min. After staining with 50 μg/ml propidium iodide, cell cycle analysis was performed by fluorescence flow cytometry on a FACScan machine (Beckman Instruments). For apoptotic analysis, cells were washed and stained using an Annexin V/PI double staining kit (BD Biosciences) according to the manufacturer’s protocol.

### Cell migration, invasion and wound healing assays

Cell migration and invasion were analyzed using a transwell chamber assay (Corning). For migration, cells cultured with serum-free medium were added to the upper chamber and medium containing 10% fetal calf serum was added to the lower chamber. For the invasion assay, insert membranes were coated with diluted Matrigel (BD Biosciences). After culture, the insert membranes were fixed and stained with 0.1% Crystal violet. Permeating cells were visualized on an Olympus BX50 microscope (Olympus Opticol Co) and Nikon Digital Sight DS-U2 (Nikon). For the wound healing assay, cells were wounded with a pipette tip and then cultured with fresh DMEM medium containing 1% fetal calf serum. Wound closing was observed every 24 h.

### Immunofluorescence staining

Cells were cultured on cover slips for 24 h. The coverslips were then washed with PBS and fixed in 4% paraformaldehyde for 15 min at room temperature. Monolayers were washed with PBS, then permeabilized with 0.5% Triton X-100 and blocked with 5% BSA for 1 h. To visualize the cytoskeleton and nuclei, cells were stained with rhodamine phalloidin antibody (1:150, Cytoskeleton) and 4′-6-diamidino-2-phenylindole (DAPI, 0.5 μg/ml). Images were acquired using an Olympus BX50 microscope (Olympus) and a Zeiss LSM510 confocal microscope (40X oil lens; Carl Zeiss).

### Rho GTPase assay

Rho GTPases were measured using the Rho GTPases activation Assay Combo Biochem Kit (Cytoskeleton) according to the manufacturer’s instructions. Briefly, cells were washed with ice cold PBS and then lysed in ice cold lysis buffer. After quantification of protein concentrations, 650 μg of cellular extracts were incubated with 10 μg Rhotekin-RBD or PAK-PBD affinity beads. The beads were then pelleted and washed. After adding 2 × Laemmli of sample buffer, GTP-bound RhoA/Rac1/CDC42 was detected by immunoblotting.

### In vivo tumorigenesis and metastasis

Male BALB/c nude mice (Institute of Zoology, Chinese Academy of Sciences) were housed in a specific pathogen-free (SPF) environment. 1 × 10^6^ cells were subcutaneously injected into twenty 4-week-old male nude mice (five mice each group) and 2 × 10^6^ cells were intraperitoneally injected into forty 5-week-old male nude mice (ten mice each group). Tumor length (L) and width (W) were measured every 5 days with calipers and tumor volume was calculated using the equation: volume = (W + L)/2 × W × L × 0.5236 [46]. Mice were sacrificed under anesthesia 30 days after injection. Tumor grafts were fixed, embedded and stained using MELK and Ki-67 antibody (Dako, dilution 1:50) by IHC. Furthermore, peritoneal nodules were visualized under microscope.

### Statistical analysis

Results were shown as mean ± standard deviation (SD). Differences in frequency of MELK expression and the correlation with clinicopathological parameters were analyzed by the Pearsonχ^2^ test. Differences between experimental groups were assessed by the Student’s *t* test or one-way ANOVA. A two-tailed value of P < 0.05 was deemed as statistically significant. Statistical analyses were performed using SPSS 19.0 software (SPSS Inc).

## Abbreviations

MELK: Maternal embryonic leucine zipper kinase; GC: Gastric cancer; qPCR: Quantitative polymerase chain reaction; IHC: Immunohistochemistry; IFC: Immunofluorescence; FCM: Flow cytometry; 3′UTR: 3′untranslated region.

## Competing interests

The authors declare that they have no competing interests.

## Authors’ contributions

TD, BL, ZZ and LS conceived and designed this work. TD, YQ, JL, HL, and QZ performed experiments and analyzed data. TD, BL, MY and CL interpreted the data and wrote the manuscript. All authors read and approved the final manuscript.

## Supplementary Material

Additional file 1: Figure S1 Effects of MELK knockdown (A) and overexpression (B) on apoptosis induced by 5-FU. Cells were treated with 0, 2, 4, 8 μg/ml of 5-FU, and apoptosis was examined by flow cytometry. Click here for file

Additional file 2: Figure S2 Effects of MELK knockdown and overexpression on cell proliferation and cell cycle progression in vitro. A and B, Cell proliferation was measured using the CCK-8 assay. MELK knockdown significantly suppresses NCI-N87 cell proliferation (**P < 0.01) and MELK overexpression slightly promotes SGC7901 cell proliferation (*P < 0.05). C, D, E and F, Cell cycle progression was monitored by flow cytometry (*P < 0.05, **P < 0.01). Click here for file

Additional file 3: Figure S3 Effects of MELK knockdown and overexpression on NCI-N87 and SGC7901 cell migration in vitro. A and B, Cell migratory ability was measured by a wound healing assay. The wound areas were measured by Image J software. These data are shown as mean ± SD of three independent experiments. C and D, Analysis of relative migration (*P < 0.05, **P < 0.01). Scale bars = 200 μm (A) and 1000 μm (B). Click here for file

Additional file 4: Figure S4 Effects of MELK overexpression on the cytoskeleton and small Rho-GTPase activity. A, Images of SGC7901/MELK and control cells (200X). Scale bars = 200 μm. B, Immunostaining of phalloidin (F-actin) in SGC7901/MELK and control cells (400X). Red: F-actin; Blue: DAPI. Scale bars = 50 μm. C, Immunostaining of phalloidin (F-actin) and DAPI (nucleus) using confocal microscopy (400X). D, Small Rho-GTPase activity in SGC7901/MELK and control cells was measured by a Rhotekin-RBD or PAK-PBD pulldown assay. Data shows examples taken from one of three independent experiments. Click here for file

Additional file 5: Figure S5 MELK-siRNA suppresses FAK and paxillin phosphorylation. A and B, MELK-siRNA partially reverses the up-regulation of pY397, pY576/577, and pY925 of FAK, and pY118 of paxillin. Click here for file

Additional file 6: Figure S6 Effects of FAK inhibitor on SGC7901 cell migration and invasion. SGC7901, SGC7901/vector and SGC7901/MELK cells were treated with FAK inhibitor (10 μM); cell migration and invasion were measured after 2 h. Scale bars = 100 μm. Click here for file

Additional file 7: Figure S7 Effects of MELK on tumor growth, peritoneal spreading and metastasis in vivo. A, Photographs of tumors derived from SGC7901/vector and SGC7901/MELK cells and growth curves in nude mice (**P < 0.01; n = 5 per group). B, Average weights of tumors in nude mice (**P < 0.01). C and D, Effects of MELK knockdown on peritoneal spreading and metastasis (**P < 0.01; n = 10 per group). E, Representative photographs of immunohistochemical analysis of Ki-67 antigen and MELK protein in tumors of nude mice (original magnification, 200X). Click here for file
