# Supplementary figures and images for: Maternal embryonic leucine zipper kinase enhances gastric cancer progression via the FAK/Paxillin pathway
Source: Mol Cancer. 2014 May 4;13:100. doi: 10.1186/1476-4598-13-100 (PMC4113179; doi:10.1186/1476-4598-13-100)

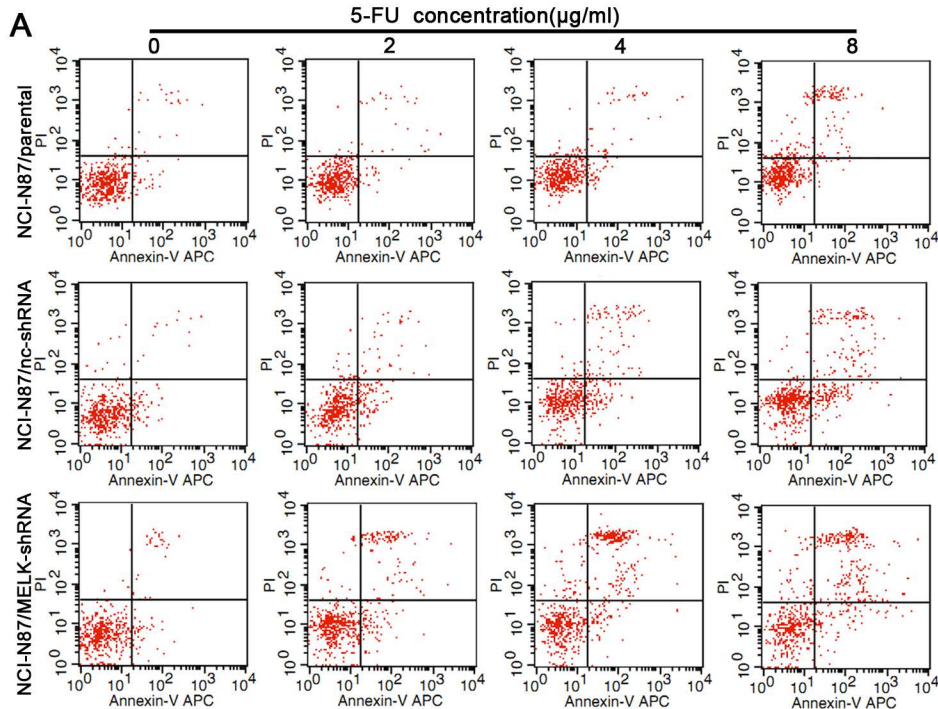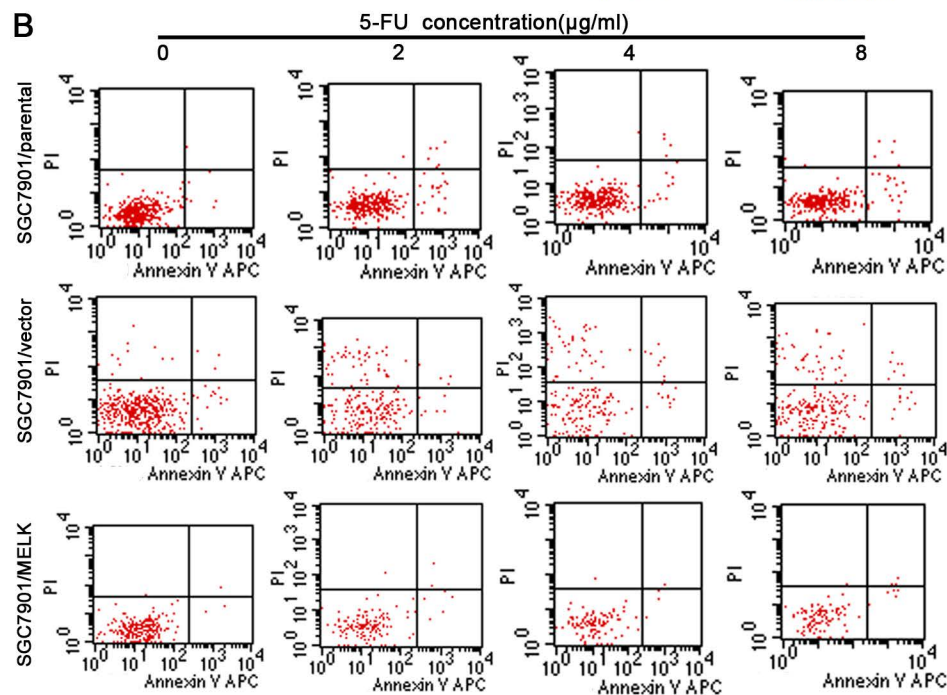

Supplement: Additional file 1: Figure S1 — Effects of MELK knockdown (A) and overexpression (B) on apoptosis induced by 5-FU. Cells were treated with 0, 2, 4, 8 μg/ml of 5-FU, and apoptosis was examined by flow cytometry. [file 1476-4598-13-100-S1.pdf]

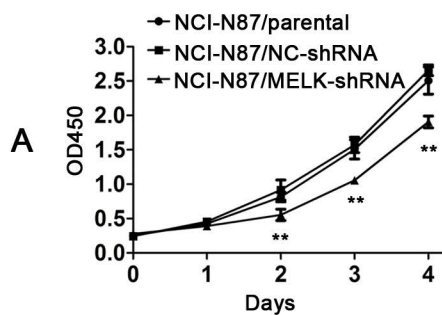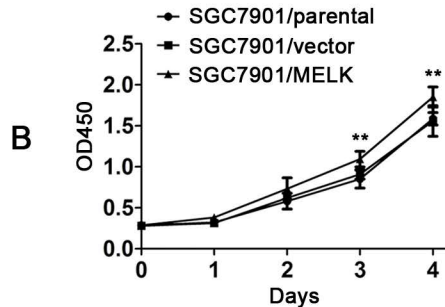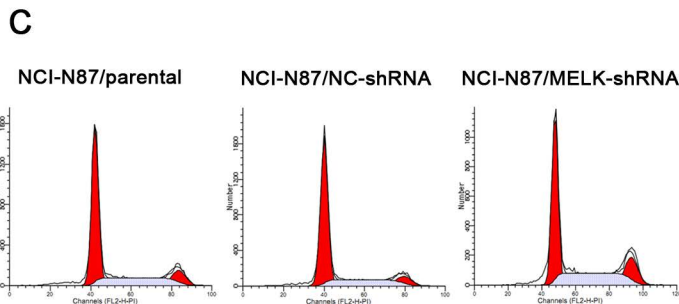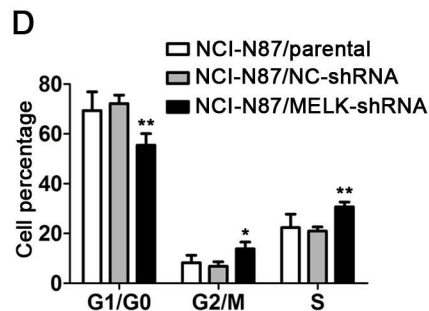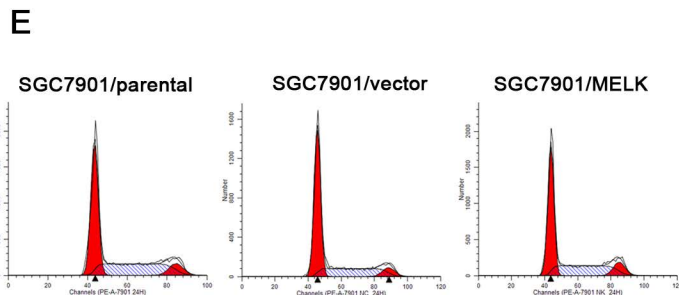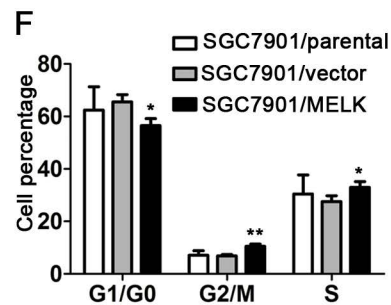

Supplement: Additional file 2: Figure S2 — Effects of MELK knockdown and overexpression on cell proliferation and cell cycle progression in vitro. A and B, Cell proliferation was measured using the CCK-8 assay. MELK knockdown significantly suppresses NCI-N87 cell proliferation (**P < 0.01) and MELK overexpression slightly promotes SGC7901 cell proliferation (*P < 0.05). C, D, E and F, Cell cycle progression was monitored by flow cytometry (*P < 0.05, **P < 0.01). [file 1476-4598-13-100-S2.pdf]

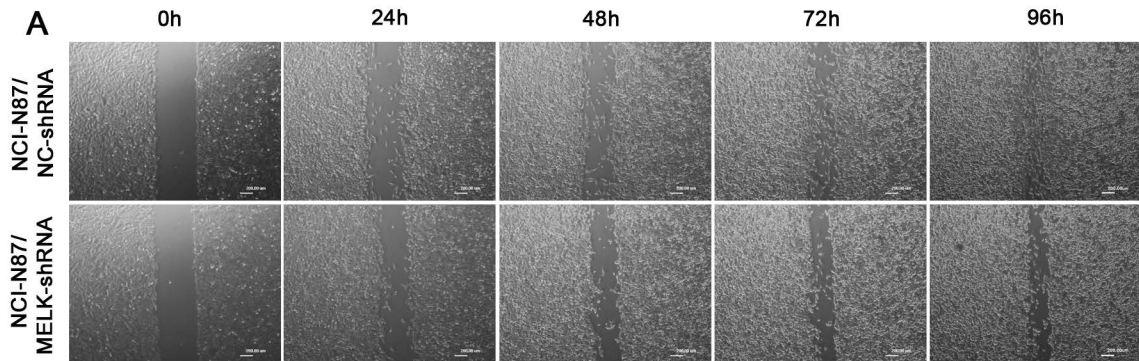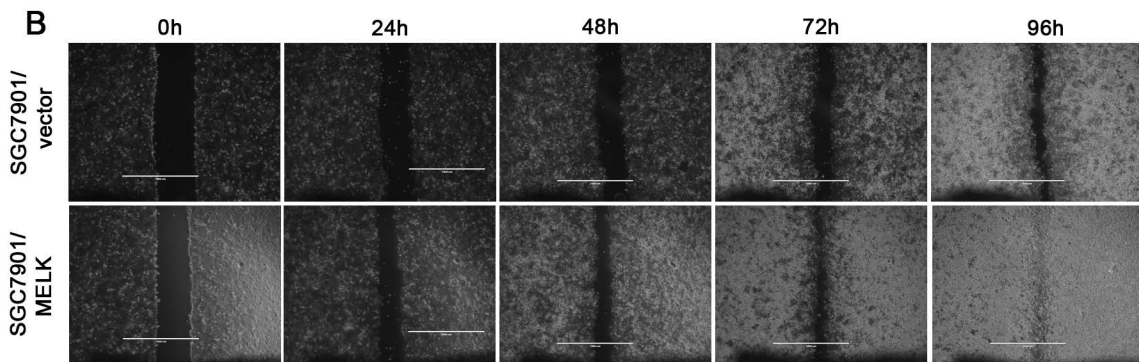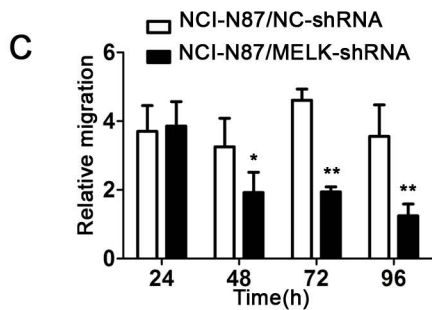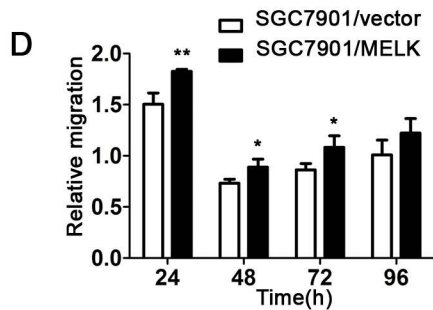

Supplement: Additional file 3: Figure S3 — Effects of MELK knockdown and overexpression on NCI-N87 and SGC7901 cell migration in vitro. A and B, Cell migratory ability was measured by a wound healing assay. The wound areas were measured by Image J software. These data are shown as mean ± SD of three independent experiments. C and D, Analysis of relative migration (*P < 0.05, **P < 0.01). Scale bars = 200 μm (A) and 1000 μm (B). [file 1476-4598-13-100-S3.pdf]

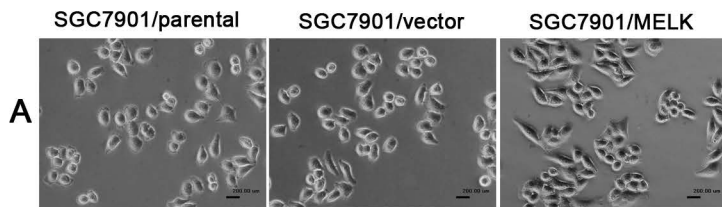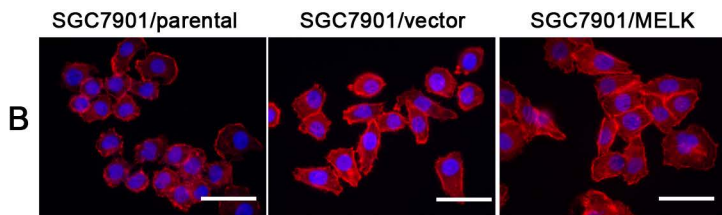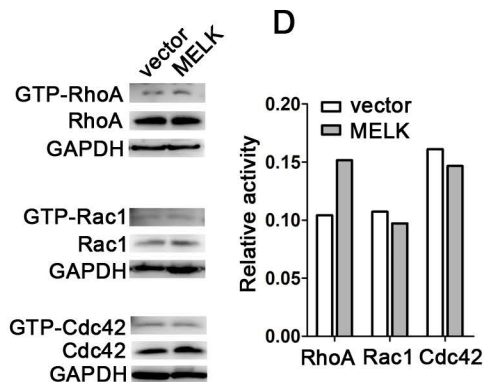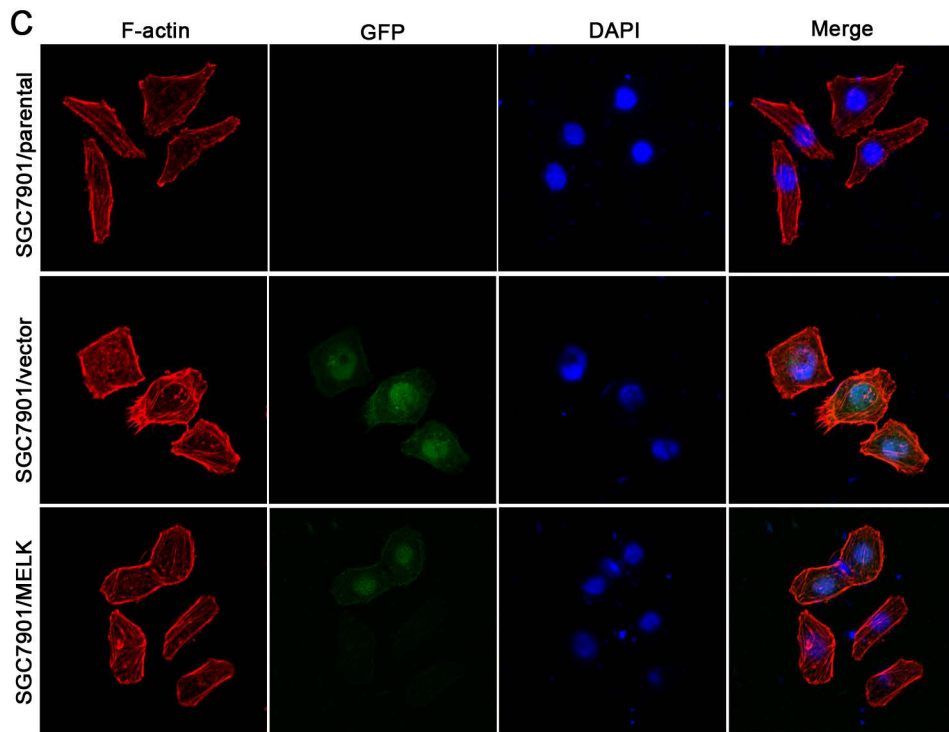

Supplement: Additional file 4: Figure S4 — Effects of MELK overexpression on the cytoskeleton and small Rho-GTPase activity. A, Images of SGC7901/MELK and control cells (200X). Scale bars = 200 μm. B, Immunostaining of phalloidin (F-actin) in SGC7901/MELK and control cells (400X). Red: F-actin; Blue: DAPI. Scale bars = 50 μm. C, Immunostaining of phalloidin (F-actin) and DAPI (nucleus) using confocal microscopy (400X). D, Small Rho-GTPase activity in SGC7901/MELK and control cells was measured by a Rhotekin-RBD or PAK-PBD pulldown assay. Data shows examples taken from one of three independent experiments. [file 1476-4598-13-100-S4.pdf]

**A**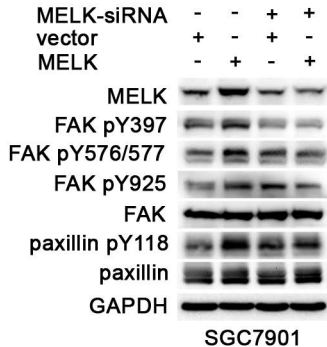**B**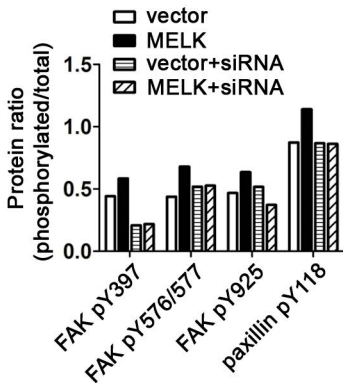

Supplement: Additional file 5: Figure S5 — MELK-siRNA suppresses FAK and paxillin phosphorylation. A and B, MELK-siRNA partially reverses the up-regulation of pY397, pY576/577, and pY925 of FAK, and pY118 of paxillin. [file 1476-4598-13-100-S5.pdf]

Migration

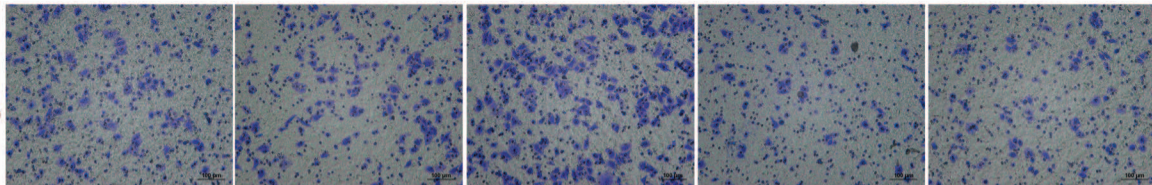

Invasion

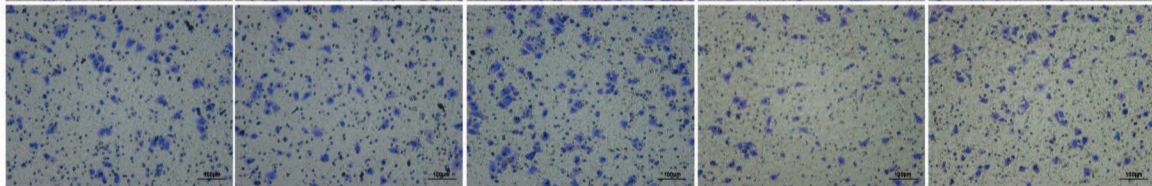

|         |   |  |   |  |   |  |   |  |   |
|---------|---|--|---|--|---|--|---|--|---|
| SGC7901 | + |  | + |  | + |  | + |  | + |
| vector  | - |  | + |  | - |  | + |  | - |
| MELK    | - |  | - |  | + |  | - |  | + |
| FAK-I   | - |  | - |  | - |  | + |  | + |

Supplement: Additional file 6: Figure S6 — Effects of FAK inhibitor on SGC7901 cell migration and invasion. SGC7901, SGC7901/vector and SGC7901/MELK cells were treated with FAK inhibitor (10 μM); cell migration and invasion were measured after 2 h. Scale bars = 100 μm. [file 1476-4598-13-100-S6.pdf]

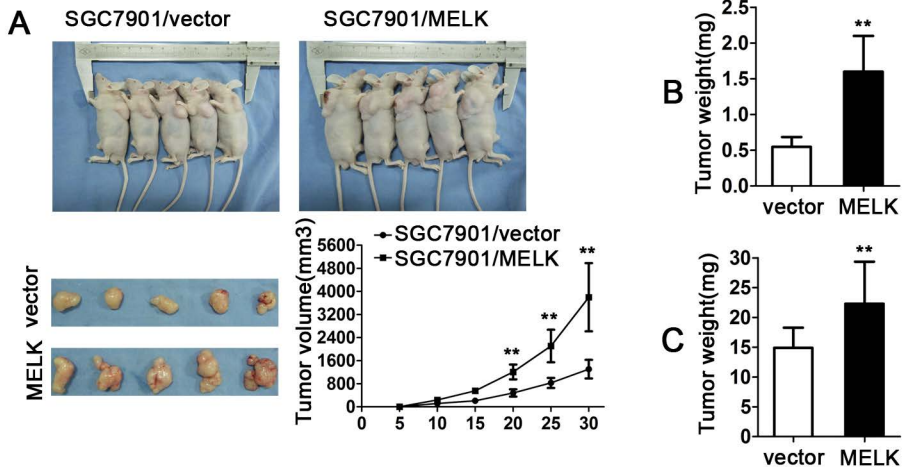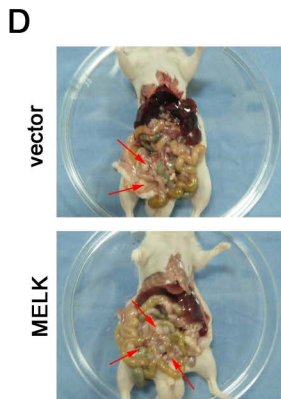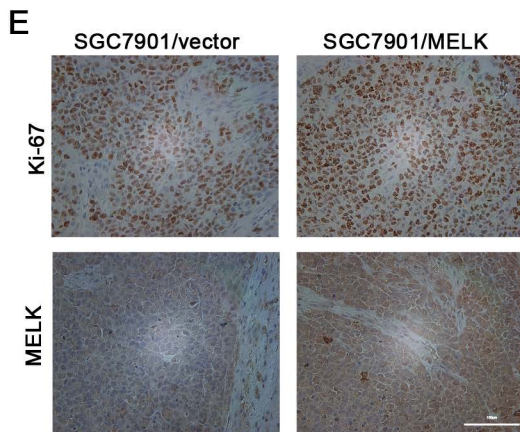

Supplement: Additional file 7: Figure S7 — Effects of MELK on tumor growth, peritoneal spreading and metastasis in vivo. A, Photographs of tumors derived from SGC7901/vector and SGC7901/MELK cells and growth curves in nude mice (**P < 0.01; n = 5 per group). B, Average weights of tumors in nude mice (**P < 0.01). C and D, Effects of MELK knockdown on peritoneal spreading and metastasis (**P < 0.01; n = 10 per group). E, Representative photographs of immunohistochemical analysis of Ki-67 antigen and MELK protein in tumors of nude mice (original magnification, 200X). [file 1476-4598-13-100-S7.pdf]
